# Shoulder tenderness was associated with the inflammatory changes on magnetic resonance imaging in patients with rheumatoid arthritis

**DOI:** 10.1038/s41598-019-55938-6

**Published:** 2019-12-20

**Authors:** Satoshi Shinagawa, Koichi Okamura, Yukio Yonemoto, Hitoshi Shitara, Takahito Suto, Hideo Sakane, Trang Thuy Dam, Tsuyoshi Tajika, Yoshito Tsushima, Kenji Takagishi, Hirotaka Chikuda

**Affiliations:** 1 Department of Orthopaedic Surgery, Graduate School of Medicine, Gunma University, Gunma, Japan; 2 Department of Diagnostic Radiology and Nuclear Medicine, Graduate School of Medicine, Gunma University, Gunma, Japan; 3 Department of Radiology, Hanoi Medical University, Hanoi, Vietnam

**Keywords:** Rheumatoid arthritis, Pain

## Abstract

The aim of this study was to assess the association between the shoulder tenderness and the inflammatory changes on magnetic resonance imaging (MRI) in the rheumatoid shoulder. Forty-one shoulders of 41 patients with rheumatoid arthritis (RA) were examined. We evaluated synovitis, erosion and bone marrow edema, by counting the numbers of each positive site, and rotator cuff tears on shoulder MRI. The association between the shoulder tenderness and the MRI findings were statistically analyzed. Twenty-three of 41 patients had tenderness in the shoulder joints. There were 20 shoulders (48.8%) with rotator cuff tear, and no significant difference was observed in the prevalence of rotator cuff tear between the tenderness group and non-tenderness group (p = 0.080). There were no significant differences in the demographic data between these two groups. In MRI findings, we found significant difference for the synovitis (p = 0.001) and bone marrow edema (p = 0.021). Synovitis was strongly associated with the shoulder tenderness (OR: 3.996, 95% CI: 1.651–9.671). Synovitis was the factor most associated with shoulder tenderness.

## Introduction

Rheumatoid arthritis (RA) is a chronic inflammatory disease characterized by synovial inflammation and bone and cartilage destruction which leads to severe disability and premature mortality. RA affects the large joints more than the peripheral joints in the later stages of the disease^1–3^. Among those large joints, 50% of RA patients have tenderness in their shoulder joints and 30% of them have decreased shoulder function one year following RA onset^4^. The destruction of shoulder joint leads to functional disabilities in their daily and work life and a decrease in the patients’ quality of life^5,6^. The shoulder joint is a complex joint which is difficult to confirm accurately the joint swelling by physical examination. Therefore, the shoulder lesions are often underdiagnosed^4^.

Structural joint damage of RA has been traditionally assessed by conventional radiography (CR) which is based on the presence of bone erosions, joint space narrowing and osteoporosis^7^. It is possible that CR images are not able to detect actual erosions of the shoulder joints in RA patients because of two-dimensional imaging.

Conversely, the examination with magnetic resonance imaging (MRI) to the shoulder joints was reported to be useful. It can not only detect rotator cuff changes but also synovitis and erosions, much earlier with a higher sensitivity. MRI can also identify bone marrow edema, which is thought to be one of the predictive factors for joint destructions^8–11^.

The shoulder joint is a complex joint which is difficult to confirm accurately the joint effusion by physical examination because of its deep location. This leads the shoulder lesions to be often underdiagnosed^11^. There have been several reports evaluating the clinical status of RA using imaging modalities, such as CR^12^, ultrasound (US)^13,14^, MRI^15,16^ and FDG-PET^17–19^. However, few reports demonstrated the association between the tenderness of shoulder and each imaging^20^.

Therefore, in this study, we investigated the associations between the shoulder tenderness and the MRI findings in patients with RA.

## Materials and Methods

### Patients

The Institutional Review Board of Gunma University Hospital, which complies with the principles of the Declaration of Helsinki, approved the protocol for this study. The informed consent was obtained from each patient and all methods were performed in accordance with the relevant guidelines and regulations. The consecutive 58 patients with RA were recruited to this study, who fulfilled the American College of Rheumatology 1987 revised criteria for RA^21^ and need to receive the anti-cytokine therapies from 2008 to 2010 in our hospital. Seventeen patients were excluded: ten declined to participate, five with renal dysfunction, and one with rheumatoid vasculitis. Thus, we investigated 41 shoulders from 41 patients. Two certified rheumatologists checked the tenderness of glenohumeral joint and recognized as the shoulder tenderness when the patients declare the tenderness. The shoulder CR and MRI images were examined on the same day. The examinations for 23 patients with tenderness were performed on the affected side, while analyses for the 18 patients without tenderness on both shoulders were performed randomly on either side.

An anteroposterior radiograph of the shoulder was obtained from each patient. The joint damage was assessed by a certified rheumatologist (Y.Y) (who had no prior knowledge of the clinical findings and results of the other imaging techniques) using the Larsen grade (0-V)^7^. For clinical evaluation, the patients were assessed using the Steinbrocker classification system^22^, the positive rates of rheumatoid factor (RF), anti-cyclic citrullinated peptide antibody (ACPA), the levels of the erythrocyte sedimentation rate (ESR), C-reactive protein (CRP), matrix metalloproteinase-3 (MMP-3), and the disease activity score (DAS) 28-ESR and DAS28-CRP^23^.

### Magnetic resonance imaging (MRI)

MRI was performed on a 1.5 T MR unit (Signa HDx, General Electric Healthcare, Milwaukee, WI, USA) using a three-channel phased array coil with the patient placed in the supine position and the adducted arm in neutral position. The following imaging sequences were used: T1, T1-weighted fast spin-echo imaging (repetition time (TR) of 465 mseconds, echo time (TE) of 10.1 mseconds, slice thickness (SL) of 4 mm, matrix size of 224 × 384 pixels, and field of view (FOV) of 180 mm) in the oblique coronal plane which was parallel to the course of the tendon of supraspinatus muscle; T2 FS, T2-weighted fast spin-echo sequences with frequency-selective fat saturation (TR of 4000 mseconds, TE of 102 mseconds, SL of 4 mm, matrix size of 224 × 384 pixels, and FOV of 180 mm) in the oblique coronal plane; Gd-DTPA T1 FS, T1-weighted fast spin-echo sequences with frequency-selective fat saturation (TR of 465 mseconds, TE of 10.1 mseconds, SL of 4 mm, matrix size of 192 × 384 pixels, and FOV of 180 mm) after intravenous administration of gadolinium-diethylenetriamine pentaacetic acid (Gd-DTPA, Magnevist, Bayer Yakuhin, Ltd., Osaka, Japan, 0.1 mmol/kg of body weight) in the axial and oblique coronal planes.

The evaluations of MRI images, such as synovitis, erosion and bone marrow edema, were defined according to previous studies^24,25^, as below. The images were evaluated using the following methods by two specialists for the shoulder joints, who are familiar with the images of shoulder joints and were blinded to the clinical status and CR findings.

**Figure 1:**
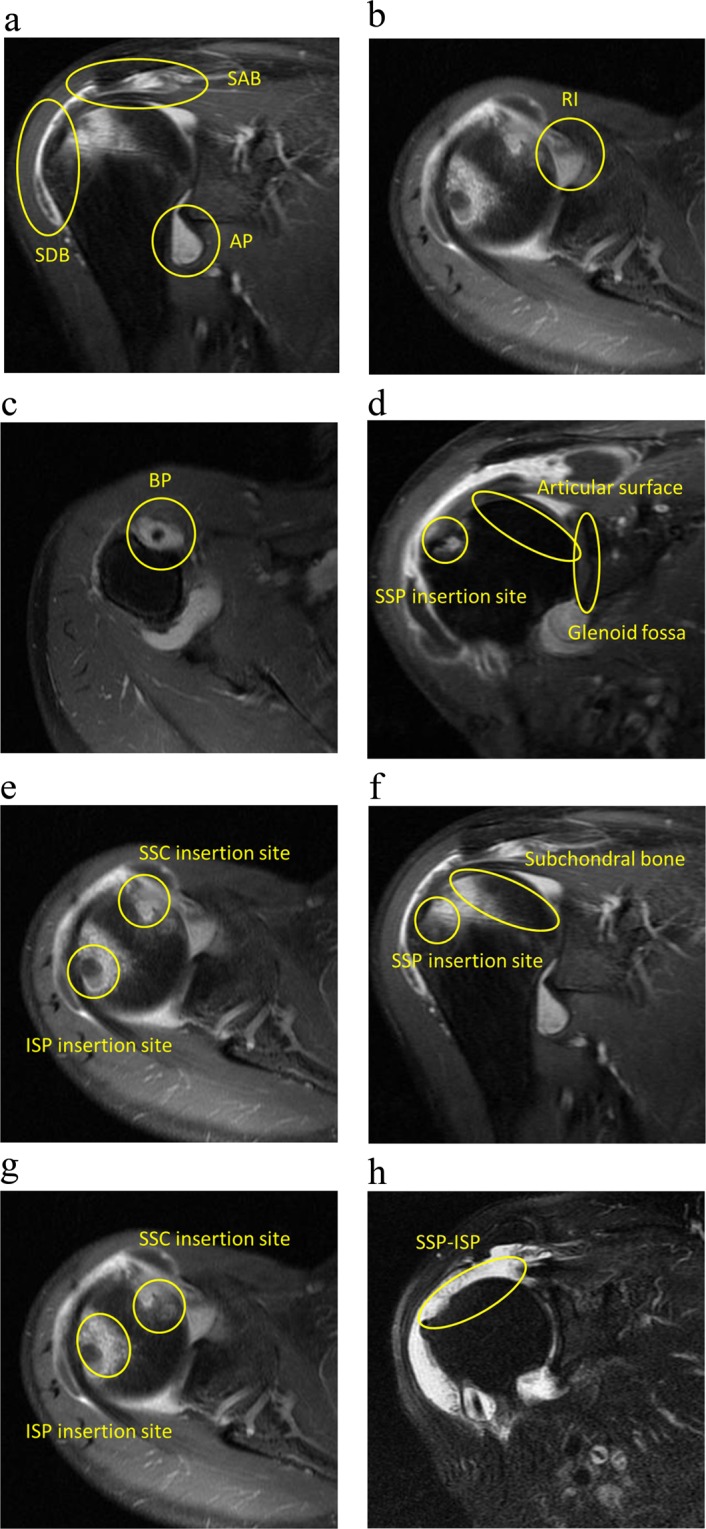
The inflammatory changes on MRI. The shoulder images are Gd-DTPA T1 FS sequences showing the synovitis (**a**–**c**), the erosion (**d,e**), the bone edema (**f,g**), and oblique coronal T2 FS showing the rotator cuff tear (**h**). Gd-DTPA: gadolinium-diethylenetriamine pentaacetic acid, T1 FS: T1-weighted fast spin-echo sequences with frequency-selective fat saturation, T2 FS: T2-weighted fast spin-echo sequences with frequency-selective fat saturation, SAB: sub-acromial bursa, SDB: sub-deltoid bursa, AP: axillary pouch, RI: rotator interval, BP: biceps pulley, SSP: supraspinatus, ISP: infraspinatus, SSC: subscapularis.

Synovitis was defined as thickened synovium (>2 mm) enhanced on Gd-DTPA T1 FS. The number of synovitis-positive sites among the sub-acromial bursa (SAB), sub-deltoid bursa (SDB), axillary pouch (AP), rotator interval (RI) and biceps pulley (BP) were counted (Fig. 1a–c).

Erosion was defined as a defect >2 mm of the cortical bone which showed low signal intensity on T1 and enhanced on Gd-DTPA T1 FS. The number of erosion-positive sites among the supraspinatus (SSP) insertion site, infraspinatus (ISP) insertion site, subscapularis (SSC) insertion site, joint surface of the humeral head and glenoid fossa were counted (Fig. 1d,e).

Bone marrow edema was defined as a lesion that showed a high signal intensity on T2 FS and a low signal intensity on T1-weighted images and enhanced on Gd-DTPA T1 FS within the trabecular bone. The number of bone edema-positive sites among the SSP insertion site, ISP insertion site, SSC insertion site and subchondral bone of the humeral head were counted (Fig. 1f,g).

The existence of rotator cuff tear of SSP-ISP was also assessed using oblique coronal T2 FS (Fig. 1h). Full-thickness and partial-thickness tears were considered as rotator cuff tear as previously described by Reinus^26^ and Teefey^27^.

### Statistical analysis

The Spearman’s rank correlation test was applied to evaluate the correlations between the Larsen grades and the existence of rotator cuff tear. The Mann–Whitney U test, the chi-square test, and Fisher’s exact test were used for comparisons between with and without tenderness groups. In addition, a logistic analysis was performed to elucidate factors associated with shoulder tenderness. IBM SPSS Statistics 25 software program (IBM Corp., Armonk, NY, USA) was used for all statistical analyses. A value of p < 0.05 was considered to be statistically significant.

## Results

The clinical characteristics are shown in Table 1.

**Table 1:** The clinical characteristics of the patients.

Characteristic	Mean ± SD (range)
Sex (male/female)	8/33
Age (years)	53.3 ± 15.5 (17–72)
Disease duration (years)	11.9 ± 12.3 (0–53)
Larsen grade (0/I/II/III/IV/V)	4/15/7/2/5/8
Steinbrocker stage (I/II/III/IV)	6/13/8/14
Steinbrocker class (1/2/3/4)	9/18/14/0
MTX: usage/dose (mg/week)	82.9%/6.9 ± 1.6 (4.0–10.0)
PSL: usage/dose (mg/day)	63.4%/4.1 ± 2.1 (1.0–10.0)
RF positivity	68%
ACPA positivity	79%
ESR (mm/h)	60.0 ± 30.8
CRP (mg/dl)	2.3 ± 2.5
MMP-3 (ng/ml)	273.2 ± 275.5
DAS28-ESR	5.4 ± 1.2
DAS28-CRP	4.5 ± 1.1

MTX: methotrexate, PSL: prednisolone, RF: rheumatoid factor, ACPA: anti-cyclic citrullinated peptide antibody, ESR: erythrocyte sedimentation rate, CRP: C-reactive protein, MMP-3: matrix metalloproteinase-3, DAS: disease activity score.

**Figure 2:**
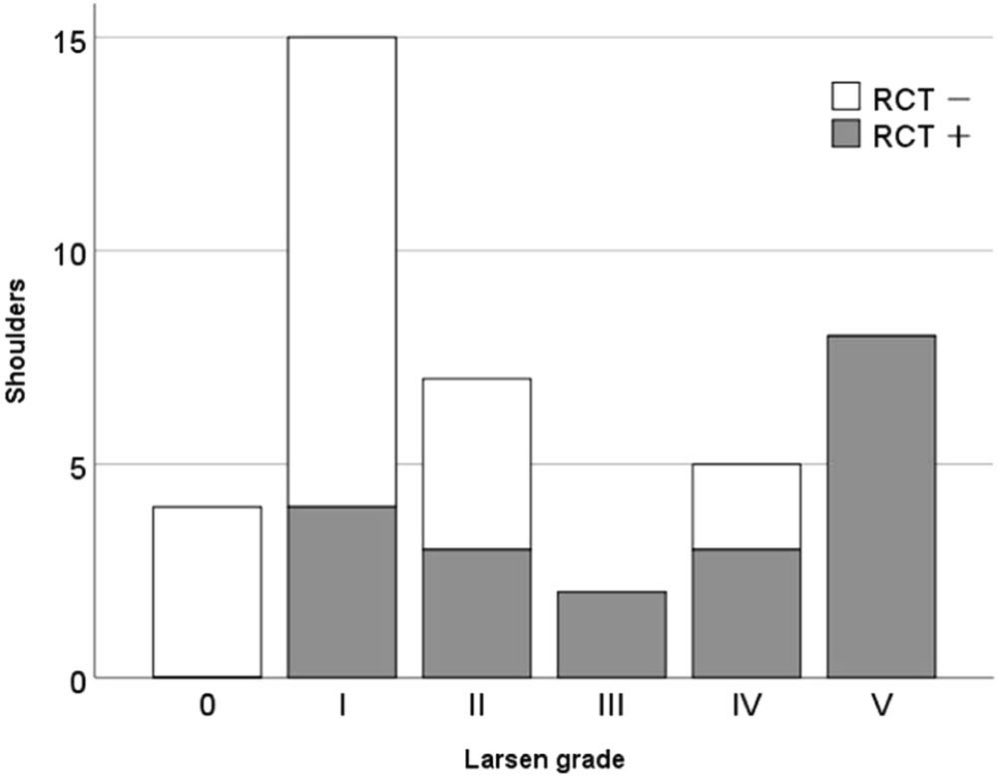
The presence and absence of rotator cuff tears for each Larsen grade. RCT: rotator cuff tear. The number of RCT is as follows: Larsen grade 0: 0/4 (0%), grade I: 4/15 (26.7%), grade II: 4/8 (50.0%), grade III: 2/2 (100%), grade IV: 3/5 (60.0%), grade V: 8/8 (100%).

Twenty shoulders (48.8%) had rotator cuff tear (14 shoulders were in tenderness group, and 6 shoulders in the non-tenderness group). The prevalence of rotator cuff tear with each Larsen grade is shown in Fig. 2. There was a positive correlation between the existence of rotator cuff tear and Larsen grade (r = 0.614, p < 0.001).

The clinical characteristics and MRI findings were compared between patients with and without shoulder tenderness (Table 2). There were no significant differences in demographic data between those two groups. The number of positive sites based on the MRI findings in the groups with/without shoulder tenderness were as follows: synovitis; 3.0 ± 1.4/0.7 ± 1.0, erosion; 3.0 ± 1.6/2.3 ± 1.3 bone marrow edema; 1.9 ± 1.3/1.2 ± 1.4. There were significant differences between the two groups for synovitis positive sites (p < 0.001) and bone marrow edema positive sites (p = 0.021).

**Table 2:** The differences in the background factors and MRI findings in the patients with and without shoulder tenderness.

	Shoulders with tenderness n = 23	Shoulders without tenderness n = 18	p-value
**Background**			
Sex (male/female)	6/17	2/16	0.213
Age (years)	54.6 ± 16.0 (17–72)	52.6 ± 15.6 (18–71)	0.762
Disease duration (years)	14.0 ± 14.2 (0.6–53.0)	8.6 ± 8.6 (0.6–30.0)	0.378
Steinbrocker stage (I/II/III/IV)	2/7/3/11	4/6/5/3	0.152
Steinbrocker class (1/2/3/4)	3/11/9/0	6/7/5/0	0.293
Larsen grade (0/I/II/III/IV/V)	1/8/2/2/4/6	3/7/5/0/1/2	0.176
use of MTX	83.3%	83.3%	1.00
dose of MTX (mg/week)	6.7 ± 1.6 (4.0–10.0)	7.3 ± 1.6 (4.0–10.0)	0.285
use of PSL	62.5%	66.7%	0.780
dose of PSL (mg/day)	4.6 ± 2.4 (1.0–10.0)	3.6 ± 1.5 (1.0–5.0)	0.317
ESR (mm/h)	59.1 ± 34.0	63.7 ± 28.4	0.430
CRP (mg/dl)	2.6 ± 2.8	2.3 ± 2.6	0.948
MMP-3 (ng/ml)	232.4 ± 220.6	330.8 ± 336.0	0.193
RF positivity	65.2%	66.7%	0.923
ACPA positivity	81.8%	76.5%	0.682
DAS28-ESR	5.6 ± 1.3	5.2 ± 1.0	0.291
DAS28-CRP	4.8 ± 1.2	4.3 ± 1.0	0.121
**MRI findings**			
Rotator cuff tear	62.5%	33.3%	0.080
Synovitis	3.0 ± 1.4	0.7 ± 1.0	<0.001*
(0/1/2/3/4/5)	(2/1/5/7/5/3)	(10/4/3/1/0/0)	
Erosion	3.0 ± 1.6	2.3 ± 1.3	0.101
(0/1/2/3/4/5)	(2/1/7/3/4/6)	(1/4/6/4/2/1)	
Bone edema	1.9 ± 1.3	1.2 ± 1.4	0.021*
(0/1/2/3/4)	(5/3/6/6/3)	(10/3/2/2/1)	

MTX: methotrexate, PSL: prednisolone, RF: rheumatoid factor, ACPA: anti-cyclic citrullinated peptide antibody, ESR: erythrocyte sedimentation rate, CRP: C-reactive protein, MMP-3: matrix metalloproteinase-3, DAS: disease activity score, MRI: magnetic resonance imaging. The Mann–Whitney U test, the chi-square test, and Fisher’s exact test were used for comparisons between the two groups. *Indicates a significant difference (p < 0.05).

The existence of shoulder tenderness was taken as a dependent variable and the numbers of sites detected as synovitis, erosion, bone marrow edema, and rotator cuff tear were used as explanatory variables in the logistic regression analysis. The most strongly associated factor with shoulder tenderness was synovitis detected with MRI (OR: 3.996, 95% CI: 1.651–9.671) (Table 3).

**Table 3:** The association between the inflammatory changes and shoulder tenderness.

	Odds ratio	95% CI	p-value
Synovitis	3.996	1.651–9.671	0.002*
Rotator cuff tear	1.938	0.135–27.790	0.626
Bone marrow edema	1.316	0.576–3.010	0.515
Erosion	0.677	0.281–1.636	0.386

CI: confidence interval, *indicates a significant difference according to the logistic regression analysis (p < 0.05).

In the non-tenderness group, 27.8% and 22.2% had synovitis at SAB and RI. On the other hand, in the tenderness group, 82.6% had synovitis at SAB, 52.2% at SDB, 65.2% at AP and RI. The incidences of all sites about synovitis in the tenderness group were higher than in the non-tenderness group. Significant differences between two groups for synovitis positive sites at SAB, SDB, AP and RI were seen (p = 0.001, p = 0.001, p = 0.001, p = 0.007, respectively) (Table 4).

**Table 4:** The location of synovitis on MRI.

		tenderness group n = 23	non-tenderness group n = 18	p value
Synovitis positive	SAB (%)	82,6	27.8	0.001*
	SDB (%)	52.2	5.6	0.001*
	AP (%)	65.2	11.1	0.001*
	RI (%)	65.2	22.2	0.007*
	BP (%)	26.1	5.6	0.112

SAB: sub-acromial bursa, SDB: sub-deltoid bursa, AP: axillary pouch, RI: rotator interval, BP: biceps pulley. *Indicates a significant difference according to Fisher’s exact test (p < 0.05).

The intra-rater reliability for synovitis, erosion and bone marrow edema were 0.977, 0.877, and 0.879 and inter–rater reliability for those were 0.939, 0.902, and 0.918.

## Discussion

In this study, we qualitatively evaluated the shoulder joints with RA patients using MRI and investigated the association between shoulder tenderness and MRI findings. We counted the number of inflammation sites on shoulder MRI which were positive for synovitis, erosion and bone marrow edema in reference to the OMERACT-RAMRIS scoring system^24^ and the Nagasaki score^28,29^. Then, we investigated these inflammatory changes by MRI and examined their associations with shoulder tenderness. Our results indicated that synovitis would highly associate with shoulder tenderness in RA.

Synovitis and erosions can be detected more readily by MRI than CR, and bone marrow edema can only be defined by MRI^30^, MRI is useful in locating RA lesions which are undetectable by CR and considered to lead to the future joint destructions^31–34^. In our study, on the even early stage of Larsen grades, some patients had high synovitis and bone marrow edema scores on MRI, independently of severity of RA.

The regression analysis revealed that the most strongly associated factor with shoulder tenderness was synovitis detected by MRI. In US study, Hirata *et al*.^20^ reported that lower concordance of shoulder tenderness and US-detected synovitis in RA. The difference between these two modalities are the possible sites for the detection of synovitis. When evaluated with MRI, the multiple detections of shoulder synovitis are more capable than the US, and this might lead to the high concordance in the MRI. In the clinical situation, synovitis should be treated intensively to prevent the future damage of the joints. Therefore, it might be suggested that the physician should consider examining the shoulder joints with MRI to check whether there is synovitis if the patients have long-lasting tenderness at shoulder joints.

The additional benefits of utilizing MRI in RA patients are the screening and detection of soft tissue damages, such as rotator cuff tears. The causes of rotator cuff tears in RA patients are not only increased age and traumatic injuries but chronic inflammation near the attachments of rotator cuffs.

Neer *et al*.^35^ found 42.0% (29/69) of rheumatoid shoulders had a complete tear of rotator cuff at total shoulder arthroplasty. The patients in their study could not use the methotrexate (MTX) and other biological disease-modifying anti-rheumatic drugs (DMARDs) since it was possible after their study that the MTX and the biological DMARDs could be administered to RA patients. In our study, although the MTX was administered to over 80% of the patients, 48.8% of our population had rotator cuff tears. These might be explained by the high disease activity of the patients in our study since they were just before the treatment of biological DMARDs. Additionally, these two rates are higher than the general population (20.7%)^36^.

Regarding the rotator cuff tears and Larsen grades, some previous studies have demonstrated that rotator cuff was the more thinning and torn in the more advanced stage of the Larsen grades^37–39^. Conversely, other reports have reported that there was no significant association between the Larsen grade and the rate of rotator cuff tears^40^. Our result was in agreement with the latter. Further research should be planned to reveal the overview of the rotator cuff tears in patients with RA, especially in the era of MTX and biological DMARDs.

There are some limitations to this study. First, this study was a retrospective cross-sectional study. Second, the patient group in this study tended to have high disease activity since they needed to receive biological therapies. Third, this study is cross sectional study and there was no information about the disappearance of the tenderness along with the treatment. Further longitudinal study is needed.

The destruction of the shoulder joint in RA patients occurred gradually, and resulting in decreased function^41^. To avoid the joint destruction, early detection of inflammation in the shoulder joints is necessary. From this study, when the shoulder tenderness is found, the imaging examination to evaluate synovitis in that joint should be considered.

## Conclusion

We evaluated synovitis, erosion, bone marrow edema and rotator cuff tear on shoulder MRI in RA patients and analyzed the association of these findings with the shoulder tenderness. Synovitis was the factor most associated with shoulder tenderness.

## Data Availability

Data sharing is not applicable to this article.
